# Variation of Reproductive Traits and Female Body Size in the Most Widely-Ranging Terrestrial Reptile: Testing the Effects of Reproductive Mode, Lineage, and Climate

**DOI:** 10.1007/s11692-013-9247-2

**Published:** 2013-08-01

**Authors:** Evgeny S. Roitberg, Valentina N. Kuranova, Nina A. Bulakhova, Valentina F. Orlova, Galina V. Eplanova, Oleksandr I. Zinenko, Regina R. Shamgunova, Sylvia Hofmann, Vladimir A. Yakovlev

**Affiliations:** 1 Department of Biology, Institute of Integrated Sciences, University of Koblenz-Landau, Universitätsstr. 1, 56070 Koblenz, Germany; 2 National Research Tomsk State University, prosp. Lenina 36, 634050 Tomsk, Russia; 3 Institute of Biological Problems of the North, Far East Division, Russian Academy of Sciences, 685000 Magadan, Russia; 4 Zoological Research Museum, Moscow M.V. Lomonosov State University, Bolshaya Nikitskaya 6, 125009 Moscow, Russia; 5 Institute of Ecology of the Volga River Basin, Russian Academy of Sciences, 445003 Togliatti, Russia; 6 Museum of Nature, Karazin Kharkiv National University, vul. Trinklera 8, 61022 Kharkiv, Ukraine; 7 Dvorichansky National Park, Dvorichna, 62701 Kharkiv Region, Ukraine; 8 Surgut State University, ul. Energetikov 14, 628400 Surgut, Russia; 9 Institute of Clinical Molecular Biology, Christian Albrecht University, 24105 Kiel, Germany; 10 Altai State Biosphere Reserve, 649000 Gorno-Altaisk, Russia

**Keywords:** Geographic variation, Lizards, Reproductive output, Reproductive mode, Maternal body size, Offspring size

## Abstract

The European common lizard, *Zootoca vivipara*, is the most widespread terrestrial reptile in the world. It occupies almost the entire Northern Eurasia and includes four viviparous and two oviparous lineages. We analysed how female snout-vent length (SVL), clutch size (CS), hatchling mass (HM), and relative clutch mass (RCM) is associated with the reproductive mode and climate throughout the species range and across the evolutionary lineages within *Z. vivipara*. The studied variables were scored for 1,280 females and over 3,000 hatchlings from 44 geographically distinct study samples. Across the species range, SVL of reproductive females tends to decrease in less continental climates, whereas CS corrected for female SVL and RCM tend to decrease in climates with cool summer. Both relationships are likely to indicate direct phenotypic responses to climate. For viviparous lineages, the pattern of co-variation between female SVL, CS and HM among populations is similar to that between individual females within populations. Consistent with the hypothesis that female reproductive output is constrained by her body volume, the oviparous clade with shortest retention of eggs in utero showed highest HM, the oviparous clade with longer egg retention showed lower HM, and clades with the longest egg retention (viviparous forms) had lowest HM. Viviparous populations exhibited distinctly lower HM than the other European lacertids of similar female SVL, many of them also displaying unusually high RCM. This pattern is consistent with Winkler and Wallin’s model predicting a negative evolutionary link between the total reproductive investment and allocation to individual offspring.

## Introduction

Quantity and quality of the offspring a female has produced determines her evolutionary fitness and ultimately the viability of the population in general. In many animal species, particularly in ectotherms, reproductive traits are strongly influenced by maternal body size. Adult body size is also important in many biological contexts other than reproduction (e.g., Blanckenhorn 2000; Meiri 2008). Therefore, offspring body size, offspring number, and body size of reproducing females are primary targets of evolutionary and ecological studies (Stearns 1992; Roff 1992).

Reptiles, particularly lizards exhibit a pronounced variation in reproductive traits and body size within and among species, and in recent decades they have been among the model groups for studying the evolution of reproductive strategies (Vitt and Pianka 1994; Shine 2005). Maternal size, climate, and ancestry (phylogenetic history) were identified as important predictors of variation in reptilian reproductive traits across related species and/or within species (e.g., Bauwens and Díaz-Uriarte 1997; Brandt and Navas 2011; Díaz et al. 2012). Theory and some empirical studies (e.g., Qualls and Shine 1995; Sun et al. 2012) suggest that reproductive mode (egg-laying vs. live-bearing) can also influence life-history traits in reptiles.

Intraspecific variation is an important issue because it links macroevolutionary patterns to microevolutionary processes which lead to the phenotypic diversity we wish to understand. While the role of ancestry is expected to be minor at this level (but see Ashton 2004), a central problem is to estimate whether the observed differences is a purely phenotypic response (plasticity) or a response to local selection (adaptation). Although common-garden and transplant experiments allow disentangling environmental and population-specific sources of geographic variation (Ferguson and Talent 1993; Sorci and Clobert 1999; Lorenzon et al. 2001), such studies are quite laborious and usually involve only two populations. Therefore, when a predominant impact of plasticity or adaptation is revealed, the ‘*regular factor*’ effect remains confounded with the *site* effect (Massot et al. 1992; Madsen and Shine 1993; Díaz et al. 2007). Thus, it remains unclear whether the study factor (e.g., a climatic variable) is a major determinant of the variation across the species range.

Wide-ranging species present promising models for simultaneously evaluating the role of different factors shaping the phenotypic diversity, because the variation of target traits can be documented from numerous localities exhibiting diverse combinations of putative predictors. However, comprehensive range-wide studies of geographic variation in widespread species are rare, even for fundamentally important traits such as offspring size and fecundity. Specifically for reptiles, only a few studies on the intraspecific variation of reproductive traits involve multiple populations and cover large geographic areas (Forsman and Shine 1995; Gregory and Larsen 1993; Iverson et al. 1997; Díaz et al. 2012).

**Fig. 1:**
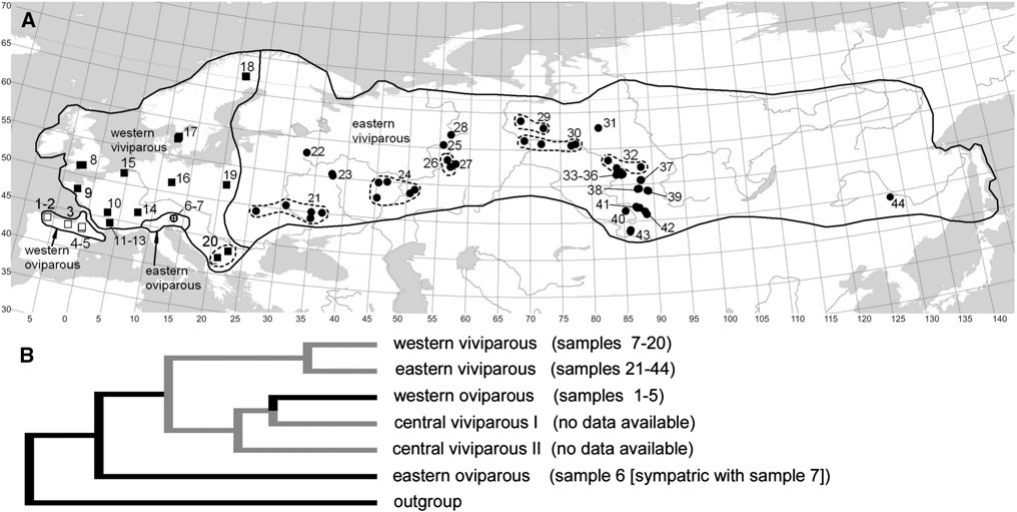
Geographic ranges of different clades of *Zootoca vivipara*, their phylogenetic relationships (after Surget-Groba et al. 2006), and our study sites. Details for study samples (1–44) are given in “Appendix”

A particularly promising candidate for such a study is the European common lizard, *Zootoca vivipara*. This species occupies much of the temperate Palaearctic (Fig. 1) and possesses the largest range among terrestrial reptiles. Moreover, *Z. vivipara* is one of the very few reptile species that occurs in both viviparous and oviparous forms (Braña and Bea 1987). Recent studies have provided an intraspecific phylogeny (Surget-Groba et al. 2006) and detailed life-history data for selected populations (e.g., Heulin 1985; Bauwens and Verheyen 1987; Massot et al. 1992). Surprisingly, no reproductive or life-history traits of this species have been studied range-wide, although some descriptive data for the eastern part of the species range have been provided (Orlova et al. 2005).

**Fig. 2:**
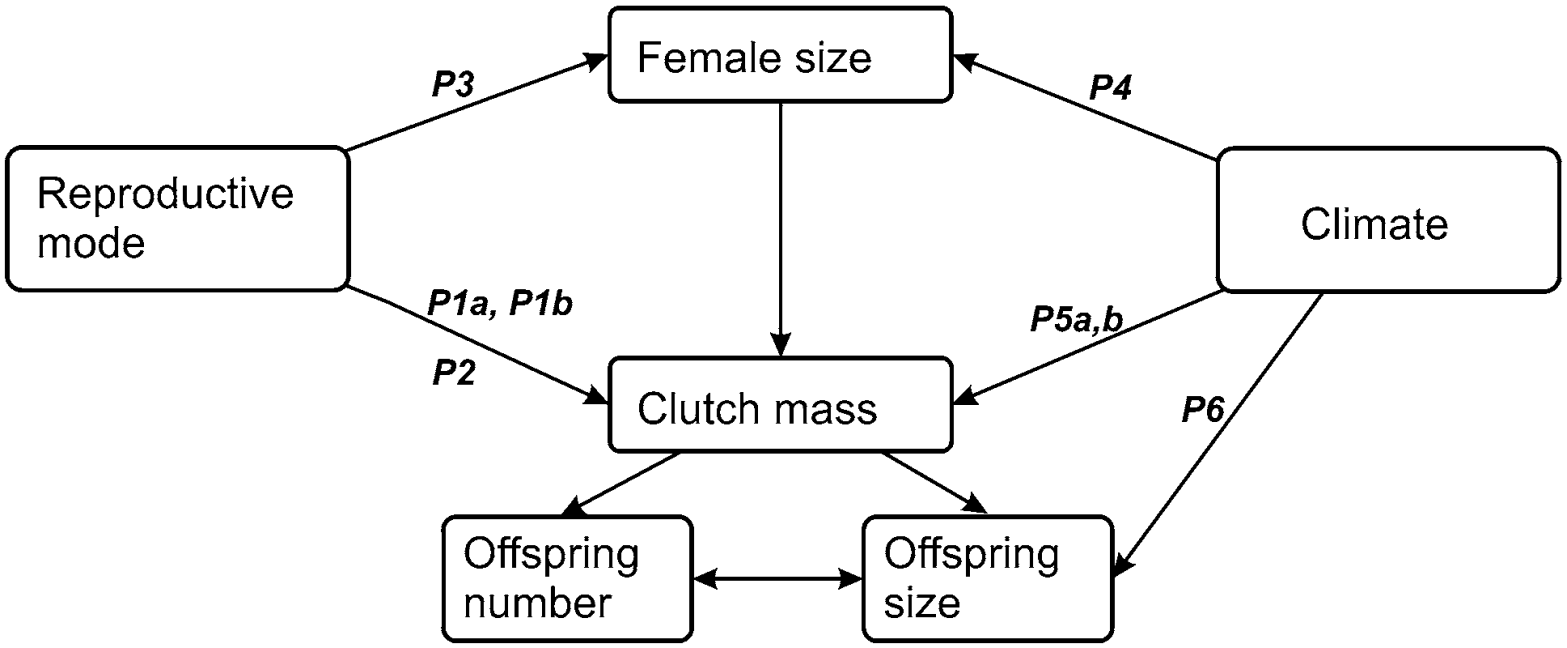
Putative relationships among dependent and independent variables, and predictions tested in our study. Note that female body size is both a dependent variable and a predictor for reproductive traits. Predictions: ***P1a***, smaller hatchling mass in viviparous versus oviparous populations; ***P1b***, smaller clutch size in viviparous versus oviparous populations; ***P2***, larger relative clutch mass in viviparous versus oviparous populations; ***P3***, larger female body length in viviparous versus oviparous populations; ***P4***, larger female body length in populations experiencing shorter versus longer activity season; ***P5***, smaller clutch size (***a***) and clutch mass (***b***) relative to female body size in populations experiencing colder versus warmer climate; ***P6***, larger hatchling mass in populations experiencing colder versus warmer climate; ***P7*** (not shown), structure of co-variation between offspring size, offspring number and female body length among population means is similar to the pattern among individual females within populations

The present study aimed to analyse patterns of intraspecific variation in reproductive traits and body size of reproducing females of *Z. vivipara* throughout the entire species range. Specifically, we assessed the effects of reproductive mode, lineage, and two climatic variables on maternal size, offspring size, and fecundity. We also explored co-variation between these life-history variables among and within populations. Our particular expectations for phenotypic responses to the listed predictors and for the pattern of co-variation are summarized on Fig. 2 and presented in detail below.

Our major prediction was that live-bearing populations of Z*. vivipara* do not show considerable evolutionary divergence in life-history. First, this hypothesis is most parsimonious. Second, extensive experimental studies of life-history variation in *Z. vivipara* revealed predominantly plastic responses to variation in physical environments (Sorci et al. 1996; Sorci and Clobert 1999; Lorenzon et al. 2001). Generally, a comparative study cannot differentiate between genetic adaptation and phenotypic plasticity. Yet in lizard life-history, for several among-trait and trait-climate relationships the prevailing adaptive hypothesis and the prevailing plasticity-related hypothesis predict opposite patterns. Differences in reproductive strategies between viviparous and oviparous strains are predicted to be adaptive evolutionary response to physical constraints due to retention of eggs in utero.

### Reproductive Mode

The phylogenetic transition from oviparity to viviparity (i.e. progressive retention of eggs in utero throughout embryogenesis) offers an opportunity to test several predictions of life-history theory, in particular the hypothesis that female reproductive output is constrained by abdomen volume (Qualls and Shine 1995; Du et al. 2005). At later stages of the embryo development, the eggs increase substantially in their mass and volume due primarily to the uptake of water (Guillette 1982; Qualls and Shine 1995; Sun et al. 2012; and references therein). In typical oviparous species, this swelling occurs after oviposition and does not burden the female. In contrast, the viviparous female retains the swelling eggs in her oviducts until the end of their development. Thus the viviparous female not only carries her reproductive burden longer than the oviparous female, but this burden, by the same net offspring mass, is larger. As a consequence, viviparous populations are expected to show either reduced offspring size (Prediction 1a) and/or number (Prediction 1b) to balance the burden, or increased relative clutch mass (Prediction 2) to maintain the net reproductive output, or increased female size (Prediction 3). These predicted responses do not exclude one another, but each of them may have fitness costs. Only few studies compared life-history traits in conspecific populations (Qualls and Shine 1995; Smith and Shine 1997; Lindtke et al. 2010) or related species (Guillette 1982; Shine 1987; Medina and Ibargüengoytía 2010; Sun et al. 2012; Yang et al. 2012) which differ in reproductive mode. Their results showed no clearly consistent pattern. *Z. vivipara* is particularly suitable for studying life-history correlates of the evolution towards viviparity because in this species each reproductive mode is represented with at least two non-sister lineages (clades) (Surget-Groba et al. 2006) thus allowing us to disentangle the effect of reproductive mode from that of phylogeny. Moreover, the two oviparous clades exhibit different degrees of egg retention (Heulin et al. 2002). Therefore, testing the above predictions, three (rather than merely two) stages along the oviparity-viviparity axis can be compared for the target traits.

### Climate

As the maternal body size is often the major determinant of geographic variation in clutch size and sometimes offspring size (e.g., Vitt and Breitenbach 1993; Iverson et al. 1997; Kiefer et al. 2008; Díaz et al. 2012), climate can act by affecting the body size distribution of reproducing females (Fitch 1985; Shine 2005; Angilletta et al. 2006). Adolph and Porter (1996) modeled the interaction of lizard growth and maturation patterns in seasonal climates. Their model predicts a nonlinear relationship between the length of activity season and the age at the first reproduction which consequently affects the minimum and average body size of adults. In the year of life in which the warm-climate individuals enter the reproduction, the individuals from colder environments (or other environments which shorten the activity season) cannot reach an appropriate body size within the time of the season at which the reproduction is still suitable. Therefore, they invest available energy to further growth and enter reproduction in the following year but at a larger size. The emerging body size cline corresponds to Bergmann’s rule (larger body size in colder climates: Blackburn et al. 1999), but the underlying mechanism is a direct response to environmental constraint rather than genetic divergence (Adolph and Porter 1996). In *Z. vivipara*, a marginally significant Bergmann’s cline was reported for altitudinal variation within an oviparous clade in northern Spain (Rodríguez-Díaz and Braña 2012). We tested whether this trend is consistent across the whole range (Prediction 4). Although Bergmann’s clines may be adaptive under some conditions (Adolph and Porter 1996; Angilletta et al. 2004), the existence of a plausible explanation of this pattern as a direct response to climate (Adolph and Porter 1996) is an important point here. Note that for the opposite pattern (converse-Bergmann’s clines), only an adaptive explanation regarding the squamate reptiles (small-sized organisms gain heat more rapidly, and in cool environments they can use short periods of sunny time more effectively than large-bodied individuals) has been proposed (Ashton and Feldman 2003; Pincheira-Donoso et al. 2008).

Climate can also affect reproductive traits independently of female body size. Female size-adjusted reproductive output is predicted to decrease in colder climates as a direct response to reduced activity and energy acquisition opportunities under thermal constraints (Congdon 1989; Adolph and Porter 1993; Rohr 1997). We tested if clutch size and clutch mass relative to female body size decreases in colder climates (Predictions 5a and 5b) in *Z. vivipara*, the species which expands further to the north than any other reptile. Noteworthy, in lizards in general the opposite pattern, an *increase* of clutch size in colder climates, is obviously predominant (Fitch 1985; Taylor et al. 1992; Vitt and Breitenbach 1993; Rocha et al. 2002; references in these papers). Although the latter pattern was found in two viviparous species (Wapstra and Swain 2001; Rocha et al. 2002), the vast majority of the species in those studies are oviparous that often produce multiple clutches in warmer climates. Consequently, the increased clutch size in colder climates can be viewed as an adaptive evolutionary response to reduced clutch frequency (Cox et al. 2003). For some special cases a plasticity-related explanation of the latter eco-geographic trend is also possible. In arid areas, deficiency of water and, consequently, low productivity may constrain energy acquisition at lower elevations more strongly than at higher elevations, with “colder” sites actually providing more benign environment than “warmer” sites (Brandt and Navas 2011; Díaz et al. 2012). However, like for converse-Bergmann’s clines, no broadly applying hypothesis viewing *increased* clutch size as a direct response to colder climates has been erected.

The available evidence for direct effects of climatic conditions on offspring size does not seem to allow plausible predictions regarding geographic variation. For *Z. vivipara*, it has been shown that females experiencing more rainfall during gestation produce smaller hatchlings, but their daughters subsequently produce fewer but larger hatchlings (Marquis et al. 2008). In contrast, there is a well established adaptive hypothesis which predicts a shift to a reproductive strategy with fewer and larger offspring in harsh environments constraining juvenile growth (Parker and Begon 1986; Roff 1992; Johnston and Leggett 2002). In *Z. vivipara*, hatchling size in the northern and southern populations of Sweden did differ as predicted by the above theory (Uller and Olsson 2003), and we examined whether this pattern is consistent across the species range (Prediction 6).

### Co-variation of Offspring Size, Offspring Number, and Maternal Size

We compared the pattern of correlation structure between offspring size, offspring number, and maternal size among individual females within populations with that among populations. The within-population correlations tend to roughly correspond to genetic correlations, the latter being maintained by stabilizing selection to favor phenotypic integration (e.g., Arnold and Phillips 1999). Similarity between the within- and among-population correlation structure (Prediction 7) is expected if genetic differentiation between study populations is negligible or largely due to stochastic processes (Revell et al. 2007 and references therein). Directional selection can break the within-population correlations, however, so that considerable adaptive divergence may often be associated with substantial differences between the within- and among population patterns (cf. Merilä and Björklund 1999; Revell et al. 2007). Thus, the above comparison provides additional indirect test of our major hypothesis.

## Materials and Methods

### Study Species

*Zootoca vivipara* is a small (adult snout-vent length 41–77 mm), ground-dwelling, insectivorous, heliothermic lizard. It occupies nearly the whole Europe and much of the northern Asia. Compared to most other lizards *Z. vivipara* shows a high resistance to low temperatures and a low resistance to desiccation (Reichling 1957). It prefers humid habitats, mostly in the forest vegetation zone.

*Zootoca vivipara* exhibits reproductive bimodality, with both the oviparous and the viviparous mode being represented with several distinct clades (lineages): western oviparous clade (WO) occupies Cantabrian Mountains and the Pyrenees with adjacent lowland areas; the Eastern Oviparous clade (EO) occurs in northern Italy, Slovenia, and southern Austria; Western Viviparous clade (WV) inhabits nearly the whole Western Europe from France to western Scandinavia, West Carpathians, and a part of Balkans; and Eastern Viviparous clade (EV) covers the greatest, eastern part of the species range (Fig. 1); two further, apparently relic clades, Central Viviparous I and Central Viviparous II, were identified from the south of central Europe (Surget-Groba et al. 2006). Their relationships derived from mitochondrial-DNA polymorphisms (Surget-Groba et al. 2006) are shown in Fig. 1b. This pattern, particularly the basal position of EO clade and a clearly distant relatedness between the two oviparous strains, is in accordance with the karyotype and chromosome structure variation (see Arribas 2009 and Lindtke et al. 2010 for brief reviews).

The young of viviparous females are usually born in the membranes and hatch within 1–2 h after oviposition (e.g., Lorenzon et al. 2001; Vercken and Clobert 2008). Less frequently, young go out of the membrane before parturition (e.g., Kuranova and Yartsev 2012), and hatching 4–6 days after parturition was also reported (Eplanova 2009; Lindtke et al. 2010).

Eggs laid by the oviparous *Z. vivipara* females possess true eggshell but contain embryos at later developmental stages than the freshly laid eggs of most other lacertid lizard species and have a relatively short incubation period of 26–37 days (Braña et al. 1991; Heulin et al. 2002; Lindtke et al. 2010; Rodríguez-Díaz and Braña 2012).

For simplicity, and taking into account the lack of qualitative distinction between the two reproductive modes in the study species, we hereafter apply terms “oviposition”, “clutch”, and “hatchling” to all populations (instead of “parturition”, “litter”, and “newborn/neonate” usually applied for viviparous taxa).

### Samples and Characters

We summarized original and published data on the following traits: snout-vent length of reproducing females, SVL; clutch size (number of eggs per clutch), CS; mean hatchling mass per clutch, HM; post-partum female mass, PPM; clutch mass, CM, taken as a difference between the female mass shortly before oviposition and the post-partum female mass in studies on viviparous populations (Pilorge et al. 1983; Pilorge 1987; Bauwens and Verheyen 1987; this study), and as total mass of freshly laid eggs in studies on oviparous populations (Braña et al. 1991; Osenegg 1995); relative clutch mass, RCM = CM/PPM.

SVL is the primary measure of overall body size in lizards and snakes (Roitberg et al. 2011 and references therein). Body mass is generally less suitable for comparative studies as it typically varies with reproductive status, fat storage, digestive state, and state of the tail. However, just PPM (except females which miss a large part of the tail) and particularly HM are free from these faults. Moreover, HM is clearly a more suitable estimator of offspring size than SVL, because inaccuracy of body length measurement in tiny newborns is too large relative to true natural variability (Massot et al. 1992). This problem is particularly relevant to our study because the data come from different researchers and, hence, may additionally include inter-observer bias (Roitberg et al. 2011).

CS reflects the fecundity in a single reproductive bout. It is an appropriate metric for total fecundity because in the vast majority of the common lizard populations (all viviparous and the highland oviparous) females produce only one clutch per year.

The RCM metric is widely used as measure of reproductive investment in reptiles (e.g., Shine 1992) because most of them, including *Z. vivipara*, display no parental care after egg laying. RCM also estimates the physical burden the female carries out (Qualls and Shine 1995).

All data on body mass and a larger part of the clutch size data have been obtained via monitoring of gravid females which were caught from the wild and held in captivity for a few days or weeks under standard conditions (Pilorge 1987, with minor modifications in other studies e.g. Uller and Olsson 2003; Lindtke et al. 2010) until parturition. The post-partum female mass and the mass of viable offspring were measured within 24 h after parturition. The female mass before parturition was recorded within the last 1–3 days of pregnancy. As manipulating of the near-term females is potentially stressful for them or their offspring, the latter trait was recorded in a smaller fraction of the females in our own studies.

A part of the clutch size data was obtained by counting oviductal eggs, embryos or (a minor fraction of data) follicles in terminal phases of vitellogenesis on autopsied females, along with measuring maternal SVL. These data come from museum samples or from previous studies mostly performed for parasitological monitoring (Kuranova et al. 2011). No animals have been sacrificed for the present study.

Additional individual-based data on the CS and maternal SVL were extracted from published scatterplots (e.g., Pilorge et al. 1983) or tables (Juszczyk 1974). Furthermore, we implemented in our analyses published mean values and other summary statistics for the relevant characters. We applied the following inclusion criteria for data from literature: (1) geographic origin of the study sample is clearly defined; (2) the sample size and some metric for variation (SD, SE or at least extreme values) are given, along with mean values; (3) data for at least two study traits are available for a given sample.

Since non-viable offspring tend to be lighter than the viable hatchlings (Massot et al. 1992) these were excluded from computations of hatchling mass. We only considered non-viable offspring when estimating clutch size to avoid a bias with the data coming from autopsied females. Deducing from extensive clutch size data on a *Z. vivipara* population in southern France (Eizaguirre et al. 2007), exclusion of non-viable offspring would result in quite minor decrease (0.07–0.20) of mean values; this bias is negligible as compared to the studied geographic variation (e.g., Orlova et al. 2005).

In total, data from 1,280 females/clutches and over 3,000 hatchlings from 76 localities across the species range were summarized (“Appendix”).

Climatic data (monthly mean minimum and maximum temperatures, and monthly mean precipitation) for the 76 study localities were obtained through the WorldClim database version 1.4, which is based on weather conditions recorded from 1950 to 2000. The spatial resolution is approximately 900 m × 900 m for Central Europe and somewhat lower for the other Eurasian regions (Hijmans et al. 2005). For few sites located in mountain regions the altitude value provided by the WorldClim deviate substantially (200–500 m) from the value given for the corresponding study sample in the original citation (apparently due to a bias in the reported coordinate values or local faults within the data base). In these cases, we corrected all temperatures for adiabatic cooling/heating (lapse rate = 0.65 °C/100 m), following the approach of Angilletta et al. (2006).

### Data Analysis

Whenever reasonable sample sizes (n > 10 females/clutches) were available we used strictly local samples, both for original and published data. Within localities, samples from different years were pooled to increase sample sizes and to apply a standard approach across the data. When local sample sizes were too small, however, we pooled them into compound samples (Fig. 1) and used in our analyses weighted means for the study traits and unweighted means for climatic variables. By pooling samples we considered (1) geographic distances, especially relative to the adjacent study samples; (2) homogeneity in terms of altitude, plant community zone, and climate; (3) lack of pronounced differences between samples for the studied traits. In few cases, data of smaller sample sizes were included if no other datasets for particular geographic region were available.

To avoid the problem of non-independence of data collected from siblings, the mean value for each clutch was used to analyze variation in hatchling mass in this and most other studies (e.g., Massot et al. 1992; Uller and Olsson 2003; Lindtke et al. 2010). Taking into account, however, that considering each hatchling as an independent observation did not significantly bias the results (Sorci and Clobert 1999; this study), population means derived with this approach were also included in our geographic variation analyses.

Considering the critique of using ratios (e.g., Packard and Boardman 1988) and regression residuals (e.g., Garcia-Berthou 2001) we controlled for confounding effects of correlated traits using ANCOVA (by placing these variables as covariates) whenever possible. We only used regression residuals to visualize a relationship of clutch size adjusted to female SVL with a climatic variable (Fig. 4) and to examine whether the revealed relationship is confounded by spatial autocorrelation. We also used a logarithm of the ratio RCM as a single dependent variable, because for many study samples the numerator (CM) or the denominator (female postpartum mass) values were not available. A lack of significant correlation between RCM and female body size in virtually all studies which tested this relationship in many lizard species (e.g., Marco et al. 1994; Qualls and Shine 1995; Olsson and Shine 1997; Tinkle et al. 1993; Wapstra and Swain 2001; Chamaillé-Jammes et al. 2006; Stuart-Smith et al. 2007; but see Pilorge et al. 1983) could argue for relevance of this widely used character, at least for rough estimations. Noteworthy, the logarithm of another ratio of two correlated traits, population means of male size and female size, was shown to have reasonable statistical properties and suggested as an appropriate metric for sexual size dimorphism (Smith 1999).

To simultaneously analyze categorical effects (Reproductive mode, Clade nested within Reproductive mode) and continuous effects (two climatic vectors and/or phenotypic variables, see below) on the variation among population means of a target trait we used generalized linear models with normal distribution and identity-link and selected the best model using Akaike’s Information Criterion for finite samples (AICc). The best model was then rerun as general linear model to assess the overall and relative strength of different predictors.

For female body length (SVL) which we treated as independent from the reproductive variables, only climatic vectors were used as covariates. For CS the set of covariates was extended for female SVL because the CS—female SVL relationship is strong and consistent in this species (e.g., Pilorge et al. 1983; this study). For HM, the co-variation with female SVL is not that strong and consistent in lizards as the CS—female SVL relationship. However, just in *Z. vivipara*, a significant interrelationship of HM, CS, and female SVL within populations was revealed (Massot et al. 1992; this study). This pattern was also revealed in variation among confamiliar species (Bauwens and Díaz-Uriarte 1997). Thus for HM the original set of covariates included two climatic and two phenotypic variables.

To investigate the possibility that an effect of climate on a study trait inferred from above models is an artefact of isolation by distance, we next tested for a relationship between among-sample distances for a study trait, climatic vectors and geographical distances. The statistical correlation between matrices of phenetic, climatic, and geographic distances was evaluated using simple and partial Mantel tests. The software used was zt (Bonnet and Van de Peer 2002), the number of permutations was 10,000. As sample 44 was a clear outlier in terms of the geographic distances (Fig. 1), it was excluded from our Mantel tests.

A principal components analysis (PCA) was used to explore patterns of multivariate divergence among populations, and of the variability among individual females/clutches within populations, in the three basic life-history variables—offspring body size (HM), offspring number (CS), and maternal body size (SVL). Principal components were extracted from the matrix of correlations among sample means (“Appendix”) and the matrix of pooled within-group (among individual females) correlations. The latter data set included seven study samples (in total 161 females) in which the three traits were recorded on at least ten females. For among-sample variation, a separate PCA was run for (1) all samples in which the three traits were available, (2) all except the single sample of the EO clade which exhibits a distinctly high hatchling mass, and (3) for the two viviparous clades. Analysis 2 was performed to test the robustness of component structure of Analysis 1; Analysis 3 can be particularly relevant for comparing with the within-sample variation because the latter was estimated on samples belonging to just these two clades.

PCA was also used to summarize the geographic variation of climatic parameters: the 36 inter-correlated temperature and precipitation variables were reduced to a smaller set of orthogonal vectors which include a major portion of the total variation. These principal components were then used as independent variables in the partial correlation and ANCOVA procedures.

When necessary, variables were log-transformed to meet the requirements of parametric tests.

### Considering the Effects of Evolutionary Lineage

A phylogeographic study by Surget-Groba et al. (2001, 2006) provided reasonably dense covering of border areas between the major clades. Thereby, virtually all our study samples could be readily assigned to particular clades based on their geographic locations. In samples 6 and 7 which come from a contact area of several clades (Fig. 1a), all study animals were examined for mt-DNA haplotypes (Lindtke et al. 2010; W. Mayer, personal communication). Surget-Groba et al. (2001, 2006) provided phylogenetic relationships among clades (Fig. 1b), but their published data gave really scarce information on the geographical distribution of haplotypes *within* the clades, and the haplotype variation within clades is apparently little, especially within the EV clade. Therefore, following Díaz et al. (2012), we employed by clade analysis (see above) to partial out the evolutionary pathway effects. Applicability of established comparative phylogenetic procedures for the intraspecific variation, which may well include reticulated evolution, is still debatable (e.g., Stone et al. 2011; see also Díaz et al. 2012); moreover, the number of units in our study is low (four clades, three dichotomies—Fig. 1b).

### Methodological Caveats

Numerous factors unrelated to geographic variation, such as short-term fluctuations in food resources or body size distributions of reproducing females, could affect reproductive traits in a particular study sample (Fitch 1985; Shine 2005). Further biases can come from pooling data of several independent researchers. They may differ in measuring routine, type of material (living vs. preserved females), and in collecting and monitoring of gravid females. The biases from the first two factors are expected to be within 2 mm or so (Vervust et al. 2009; Roitberg et al. 2011), and that is much lower than the observed variation within and among our study samples. Animal keeping details should also be a minor caveat for this study because the vast majority of gravid females were obviously collected after ovulation, the stage at which their reproductive output is determined (Bauwens and Verheyen 1987; Uller and Olsson 2005). Finally, and most importantly, the above confounding factors are unlikely to create a regular pattern shaped by a large number of independently collected data units. Such robust patterns are considered for our discussion.

## Results

### Climatic Variation Across the Study Sites

The first axis of the principal components analysis on the climatological variables (PC1-clim) explained 57.1 % of the total variance among localities (Table 1). PC1-clim is strongly and positively loaded with all monthly temperature and precipitation parameters outside the warmest quater (Table 1); PC1-clim is highly correlated with mean annual temperature (Spearman rank correlation coefficient, *p*_*s*_ = 0.98, *n* = 44, *P* < 0.001) and mean temperature of the coldest (*p*_*s*_ = 0.96, *P* < 0.001) but not the warmest (*p*_*s*_ = 0.27, *P* > 0.09) quarter.

**Table 1:** Factor loadings and percents of trace associated with the first two principal components of among-sites variation in climatic parameters

Trait	PC1	PC 2	Trait	PC1	PC 2
tmin1	0.977	−0.012	tmin7	−0.020	0.838
tmax1	0.977	−0.018	tmax7	−0.195	0.876
prec1	0.816	−0.416	prec7	−0.237	−0.437
tmin2	0.981	−0.006	tmin8	0.499	0.751
tmax2	0.978	0.007	tmax8	0.358	0.860
prec2	0.828	−0.485	prec8	0.024	−0.639
tmin3	0.985	0.083	tmin9	0.885	0.379
tmax3	0.965	0.122	tmax9	0.778	0.511
prec3	0.799	−0.542	prec9	0.533	−0.682
tmin4	0.911	0.368	tmin10	0.968	0.190
tmax4	0.811	0.510	tmax10	0.944	0.202
prec4	0.769	−0.522	prec10	0.710	−0.572
tmin5	0.728	0.627	tmin11	0.974	0.056
tmax5	0.356	0.825	tmax11	0.981	0.061
prec5	0.581	−0.660	prec11	0.789	−0.464
tmin6	0.257	0.879	tmin12	0.973	−0.003
tmax6	−0.084	0.921	tmax12	0.981	0.001
prec6	0.261	−0.661	prec12	0.830	−0.444
			% of trace	57.06	27.84

In contrast, the second principal component (PC2-clim, 27.8 % of the total variance, Table 1) is heavily loaded just with the monthly values of the warmest season (May–August), with consistently positive loadings of temperatures and consistently negative loadings of precipitation (Table 1). PC2-clim is tightly related to the mean temperature of the warmest quarter (*p*_*s*_ = 0.94, *n* = 44, *P* < 0.001) but not the coldest quarter (*p*_*s*_ = 0.17, *n* = 44, *P* > 0.3).

### Body Length (SVL) of Reproducing Females

**Fig. 3:**
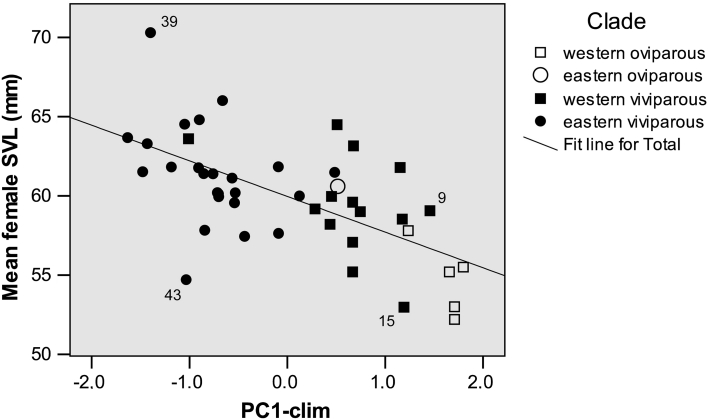
Mean snout-vent length (SVL) of reproducing females in different study samples of *Zootoca vivipara* and scores of PC1-clim which encompasses 57 % of the total climatic variation. PC1-clim is highly positively loaded with mean monthly values of minimum temperature, maximum temperature, and precipitation of all months besides the warmest quarter (see Table 1 for details). Numbers of study samples as in Fig. 1 and “Appendix”

For mean SVL of reproducing females, the best model, explaining about 40 % of the among-sample variance, included only one predictor, PC1-clim (Table 2). The relationship between the two variables is shown on Fig. 3. Mantel test revealed a significant correlation between the matrix of among-sample distances for PC1-clim with the matrix of geographic distances (*r* = 0.613; *P* = 0.023). When the effects of proximity are factored out, then SVL is still significantly associated with PC1-clim (partial Mantel test, *r* = 0.295; *P* < 0.001). Geographic proximity is poorly associated with SVL, however, when the effects of PC1-clim are factored out (partial Mantel test, *r* = −0.110; *P* = 0.052).

**Table 2:** Our best models for geographic variation in female SVL and reproductive traits of the lizard, *Zootoca vivipara*

	*df1*	*df2*	*MS*	*F*	*P*	% variance (partial eta × 100)
*Female SVL*						
Corrected model	1	42	219.25	26.38	0.000	38.6
PC1	1	42	219.25	26.38	0.000	38.6
*Clutch size*						
Corrected model	1	42	7.87	5.40	0.025	11.4
PC2	1	42	7.87	5.40	0.025	11.4
*Clutch size relative to SVL*						
Corrected model	3	40	12.01	14.52	0.000	52.1
Female SVL	1	40	27.32	33.04	0.000	45.2
PC2	1	40	15.33	18.54	0.000	31.7
Female SVL × PC1	1	40	6.73	8.14	0.007	16.9
*Relative clutch mass*						
Corrected model	2	13	0.17	6.22	0.013	48.9
Reproductive mode	1	13	0.18	6.69	0.023	34.0
PC2	1	13	0.23	8.42	0.012	39.3
*Mean hatchling mass*						
Corrected model	3	20	0.06	11.47	0.000	63.2
Reproductive mode	1	20	0.14	28.36	0.000	58.6
clad(repro)	2	20	0.04	8.60	0.002	46.2

Noteworthy, the two most divergent populations of the eastern viviparous clade—“giant” females from the eastern slope of the Kuznetsky Alatau ridge (mean SVL 70 mm, sample 39) and small-sized females from the Lake Markakol coast (mean SVL 54 mm, sample 43)—considerably deviate from the general climate-related trend (Fig. 3). Both populations are located within the Altai–Sayan Mountain Region which lies to the south–east of the West Siberian Plain.

### Clutch Size

**Fig. 4:**
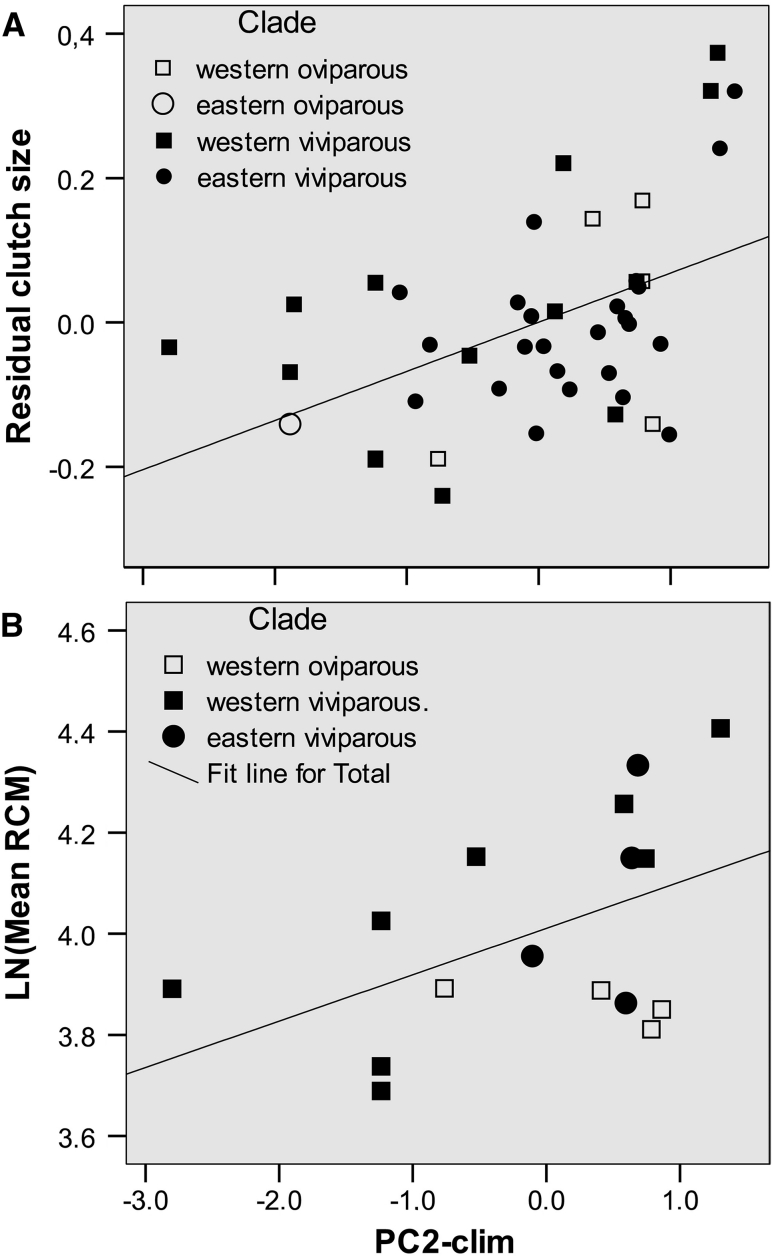
Relative fecundity (residuals of the regression of mean clutch size on mean female body length, **a**) and relative clutch mass (RCM, **b**) in different study samples of *Zootoca vivipara* plotted against scores of PC2-clim. All phenotypic variables are log_e_ transformed

Mean clutch size showed a considerable geographic variation across the species range (from 4.3 to 9.8—“Appendix”), but our best model (including PC2-clim only) explained less than 12 % of the total variance (Table 2). When the set of predictors had been extended with Female SVL the best model explained above 50 % of the total variance; besides Female SVL this model included PC2-clim and the Female SVL × PC1-clim interaction (Table 2). The relationship between relative CS (taken as residuals of the regression of mean CS on mean female SVL across study samples) and PC2-clim is shown on Fig. 4a.

Mantel test revealed no correlation between the matrix of among-sample distances for relative CS with the matrix of geographic distances (*r* = 0.03; *P* = 0.210) so that the above relationship is not an artefact of spatial autocorrelation.

### Relative Clutch Mass (RCM)

Mean RCM varies considerably among study samples, ranging from 45 to 49 % in the WO clade, 40 to 82 % in the WV clade, and 48 to 76 % in the EV clade (“Appendix”). The best model, explaining about 50 % of the among-sample variance, included Reproductive mode and PC2-clim (Table 2). When we replaced the two groups of closely located samples (11, 12, 13 and 34, 35, 36—Fig. 1) with the average values we got the same significant predictors with even a higher rate (70 %) of explained variance. The relationship between RCM and PC2-clim is shown on Fig. 4b.

Within the group of viviparous populations, Mantel test revealed no correlation between the matrix of among-sample distances for RCM with the matrix of geographic distances (*r* = −0.126; *P* = 0.127), whereas the correlation with PC2-clim was significant both with (*r* = 0.295; *P* = 0.029) and without (*r* = 0.289; *P* = 0.036) control for geographic proximity. Thus, the revealed relationship between RCM and PC2-clim is not an artefact of spatial autocorrelation.

### Mean Hatchling Mass

The best model, explaining 63 % of the total variance, included Reproductive mode and Clade (Table 2). Hatchlings of the single study sample of the EO clade are much heavier than these of all other study populations (Table 3), and hatchlings of the WO clade are heavier than those of the WV clade (ANOVA, the three clades with multiple samples: *F*_2,19_ = 4.43, *P* < 0.03; Tukey HSD test, *P* < 0.05; Table 3). Thus our results clearly demonstrate that hatchlings tend to be heavier in oviparous than in viviparous populations, and within the former group hatchlings are heavier in the EO clade.

**Table 3:** Modal stage of embryos (according to the developmental series of Dufaure and Hubert 1961) in freshly laid eggs and mean hatchling mass (average value of population means) in oviparous and viviparous clades of Zootoca *vivipara*

Clade	Stage of embryos at oviposition	Hatchling mass (mg)
Eastern oviparous	31	277 (n = 1)
Western oviparous	32–34	205 (n = 3)
Western viviparous	40	184 (n = 11)
Eastern viviparous	40	194 (n = 9)

Data for stages are from Heulin et al. (2002), Lindtke et al. (2010) (EO clade), Braña et al. (1991), Rodríguez-Díaz and Braña (2012) (WO clade), stage 40 corresponds to fully developed newborns (Dufaure and Hubert 1961). Data for hatchling mass are from “Appendix”

Within the group of viviparous populations, Mantel test revealed a significant correlation between the matrix of among-sample distances for HM with the matrix of geographic distances (*r* = 0.168; *P* = 0.031). This correlation persisted when corrected for PC1-clim (*r* = 0.211; *P* = 0.024) or PC2-clim (*r* = 0.187; *P* = 0.024).

### Co-variation Between Female SVL, Clutch Size, and Hatchling Mass

Principal components analysis (PCA) of within-sample and between-sample variation for female SVL, clutch size and hatchling mass (Table 4) revealed a pronounced similarity in correlation structure at these two levels. This similarity is particularly strong when the data set for geographic variation is restricted to the two viviparous clades (a separate considering of these two clades is relevant because they form a monophyletic unit and because the within-sample variation was estimated on samples belonging to these two clades). In both the within-sample and among-sample variations, PC1 includes 51–59 % of the total variance and is strongly associated with female SVL and clutch size, whereas PC2 (33–34 %) is heavily loaded with hatchling mass and weakly correlated with the two other traits. When the whole data set (including oviparous populations) is considered, the among-sample variation differs from the within-sample variability by a lack of the following tendencies: a positive association between hatchling size and female SVL on PC1 and a negative association between clutch size and hatchling mass on PC2 (Table 4).

**Table 4:** Factor loadings and percents of trace associated with the first two principal components of the within and among-population variation for three basic life-history parameters

	Pooled variation among individual females within samples (n = 161)	Variation among sample means		
		All samples (n = 24)	All without EO clade (n = 23)	WV + EV clades (n = 20)
*PC1*				
Female SVL	0.868	0.895	0.910	0.935
Clutch size	0.779	0.920	0.915	0.908
Newborn mass	0.417	−0.183	−0.182	0.244
% variance	51.2	56.1	56.7	58.6
*PC2*				
Female SVL	−0.024	0.231	0.126	−0.021
Clutch size	−0.447	−0.030	0.070	−0.238
Newborn mass	0.884	0.978	0.983	0.967
% variance	32.7	33.7	32.9	33.1
*PC1* *+* *PC2*				
% variance	83.9	89.8	89.6	91.7

**Fig. 5:**
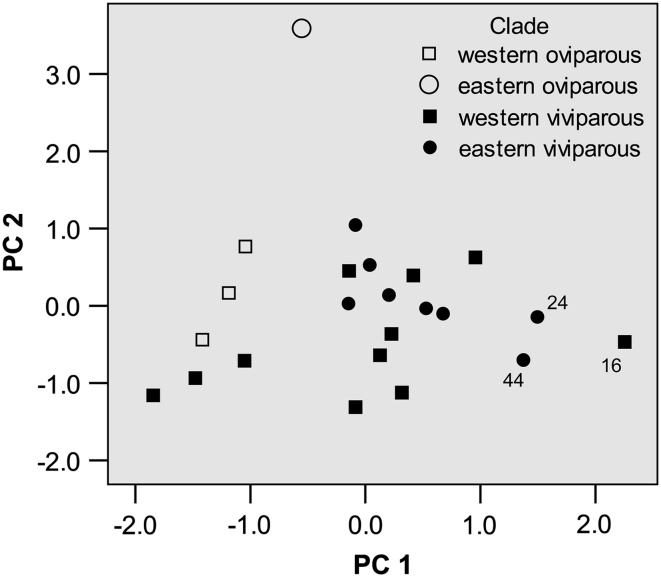
Plot of scores of the first two principal components of the variation among sample means of the three basic traits in 24 geographically distinct samples of *Zootoca vivipara*. Numbers of study samples as in Fig. 1 and “Appendix”

Figure 5 shows ordination of study samples along the first two principal components which include ca. 90 % of the considered geographic variation. As expected, the single sample of the EO clade is strongly separated from all other samples along PC2 due to its high hatchling mass (Table 3). Furthermore, the oviparous samples tend to cluster from the viviparous samples by a combination of higher values of PC2 and lower values of PC1. In the subset of viviparous samples, neither clades nor major geographic regions tend to cluster, and these which do cluster (samples 16, 24, and 44) differ strongly in their geographic origin (eastern Germany, Middle Volga, and north-eastern China, respectively).

## Discussion

Overall, our study showed a considerable amount of geographic variation in mean female body size and reproductive life-history in *Z. vivipara*. The study traits differed strongly in the set of their predictors in our analyses. Female SVL and clutch size were affected by climatic vectors, with no significant impact of reproductive mode or evolutionary lineage (clade). These effects remained significant when the effects of geographic proximity were controlled for. In contrast, mean hatchling mass was strongly affected by reproductive mode and clade (and weakly by geographic proximity), without respect to climate. The variation of RCM showed some intermediate pattern, with climate and reproductive mode and/or clade being significant predictors. Below we put the particular trait-predictor relationships into the context of other life-history studies and discuss how they relate to our special predictions (Fig. 2). The difference in the set of predictors is in line with findings from other lizard ife-history studies: in common garden experiments (Ferguson and Talent 1993) and long-term studies of a model population (Massot et al. 1992; Chamaillé-Jammes et al. 2006; Le Galliard et al. 2006), hatchling mass was the least plastic trait and showed large heritability (Le Galliard et al. 2006).

The above patterns for female size and fecundity correspond well with our principal hypothesis on a major role of plasticity in the geographic life-history variation in *Z. vivipara*. The pattern for offspring size apparently manifests evolutionary divergence between viviparous and oviparous strains. Below we put the particular trait-predictors relationships into the context of other life-history studies and discuss how they relate to our special predictions (Fig. 2).

### Reproductive Mode

A decrease of hatchling mass in the series *EO clade*—*WO clade*—*viviparous populations* corresponds well to the hypothesis that female reproductive output is constrained by abdomen volume (Qualls and Shine 1995; Du et al. 2005), and this constraint is exerted by progressive egg retention (Guillette 1982; Qualls and Shine 1995). EO clade occupies the basal position in the species phylogeny (Surget-Groba et al. 2006) and exhibits only a moderate rate of egg retention (Table 3); this clade has the heaviest hatchlings (Table 3). WO clade has a higher rate of egg retention and, respectively, lower hatchling mass (Table 3). Finally, viviparous females have still lighter hatchlings (Table 3). Note that the above pattern of among clade differences in hatchling mass does persist after correcting for variation in female SVL and clutch size, being also resistant to correcting for climate.

**Fig. 6:**
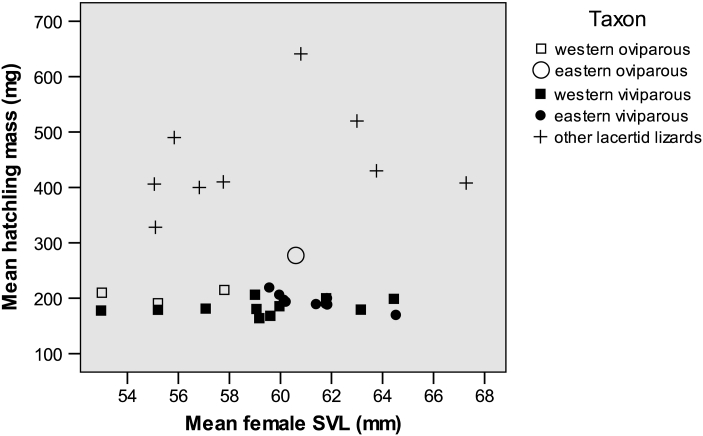
Mean hatchling mass and mean body length of reproducing females in different populations of *Zootoca vivipara* and other European species of the family Lacertidae. Data for *Z. vivipara* are from “Appendix”; data for other species are from: Arribas and Galán (2005), Castilla and Bauwens (2000), Galán (1999), Galán and Vicente (2003), Ji and Braña (2000), Ljubisavljević et al. (2007, 2012), Rúa and Galán (2003)

Although EO clade is represented by a single population in our data set, a large magnitude of its differences from the other populations (Table 3), including the parapatrically occurring viviparous population (Lindtke et al. 2010), allows us to be confident of the terminal position of EO clade in the above series. Interestingly, mean hatchling mass of EO clade (as estimated with the single study sample), being a positive outlier among the conspecific populations, is yet lower than in the other related species with comparable female SVL for which we could find relevant data (Fig. 6).

Thus, our study clearly demonstrates that in the European common lizard, a progressive extent of egg retention was accompanied by a decrease in offspring size (hatchling mass). Although this pattern of offspring size variation is in line with the ‘volume constraint hypothesis’ (Prediction 1a), this pattern was not common in previous studies. It was reported in none of the other two reproductively bimodal lizard species (Qualls and Shine 1995; Smith and Shine 1997) and in only two of the 7 lizard and snake genera in which oviparous and viviparous species were compared (Guillette 1982; Shine 1987; Medina and Ibargüengoytía 2010; Sun et al. 2012; Yang et al. 2012).

The most frequently reported pattern in the above-cited studies is a larger female size of the viviparous forms versus their oviparous counterparts (both two reproductively bimodal species; 4 of the 7 reproductively bimodal genera—*op. cit.*). This pattern is also congruent with the ‘volume constraint hypothesis’ (Prediction 3). In *Z. vivipara*, reproducing females of EO clade show smaller body length than those of WV clade collected from virtually the same site (“Appendix”). Also, populations of WO clade tend to exhibit smaller female SVL than the viviparous populations. However, the smaller female SVL of WO clade appears to be largely induced by milder climate of the region it inhabits (Fig. 3). Furthermore, the existence of a rather large-bodied oviparous population (EO clade, Fig. 3) on the one side, and remarkably small-bodied viviparous populations (samples 15 and 43, Fig. 3) on the other side, argue for a relatively moderate impact of the reproductive mode in shaping the overall geographic variation for female SVL in our study species.

The third pattern related to the ‘volume constraint hypothesis’ is a tendency of viviparous populations to show higher mean RCM than in oviparous populations (Prediction 2). In our study, this pattern is statistically significant when considering for geographic variation in PC2-clim. Considering this circumstance, a lack of data on the EO clade, and the different technique of measuring RCM in oviparous and viviparous females (see “Methods”), we qualify the present evidence as only suggestive. In another reproductively bimodal species, *Lerista bougainvillii*, mean RCM was clearly higher in the viviparous versus oviparous females (Qualls and Shine 1995), whereas the third species, *Saiphos equalis* shows an opposite tendency (Smith and Shine 1997). The above-cited between-species comparisons provide no relevant data because they actually used a clearly incomparable metric for RCM based on the total *net* mass of hatchlings.

### Climate

Mantel tests indicated that climate (PC1-clim or PC2-clim) but not geographic proximity, is related to geographic variation in female SVL, relative fecundity, and RCM.

The first principal vector of the inter-locality variation of monthly means of minimal temperature, maximal temperature, and precipitation (PC1-clim, Table 1) can be interpreted as an index of climate mildness. Its negative values correspond to more continental climates with a long winter season and a short (even though sometimes warm) summer, whereas its positive values correspond to benign climates with a short winter and a prolonged period with higher (even though not quite high) temperatures. Therefore, PC1-clim is a good proxy for the length of activity season, and its negative correlation with mean body length (SVL) of reproducing females is in line with Adolph and Porter’s (1996) model. As PC1-clim is tightly associated with mean annual temperature the revealed body size cline corresponds to Bergmann’s rule (Prediction 4). Although Bergmann’s clines were found in many squamate species (e.g. Wapstra and Swain 2001; Rocha et al. 2002 and references therein; Leaché et al. 2010), about 70 % of lizard and snake species exhibit converse-Bergmann’s clines (Ashton and Feldman 2003). Remarkably, even in closely related species, opposite body size clines can occur (Ashton 2001; Sears and Angilletta 2004). In this context it does not seem unreasonable to assume that in a wide-ranging species, multiple independent divergences along similar climatic gradients may differ in their underlying mechanisms. Depending on the relative occurrence of the two patterns in the data set, a trend distorted by outliers (this study) or a lack of an overall trend can be revealed. In any event, the existence of strongly deviating populations in the present study indicates that factors other than the length of activity season (Adolph and Porter 1996) contribute to shaping the geographic body size variation in the study species. Obviously, even the age at maturity is not always the primary determinant of mean body size of reproducing females. In northwestern Europe, mean age at first reproduction is ca. 1.5 years in Paimpont (Heulin 1985), and ≥2 years in Kalmthaut (Bauwens and Verheyen 1987). Opposite to the prediction of Adolph and Porter’s (1996) model the former population exhibits a higher mean SVL of gravid females than the latter one (Fig. 3, Samples 9 and 15, respectively).

The second principal vector of the studied climatic variation (PC2-clim, Fig. 2/Table 1) characterizes the warmest quarter, its lower values being associated with cool and wet summer, and its higher values with warm and relatively dry (even though sometimes short) summer. The positive correlations of PC2-clim with both the SVL-adjusted clutch size and RCM imply that the decrease of relative fecundity in colder climates revealed in *Z. vivipara* is due to a shift in the total reproductive output rather than in the position of the offspring size-number tradeoff (see also the next paragraph). This pattern, which was also found in another viviparous lizard (Rohr 1997), is consistent with Prediction 5.

We found no significant relationship between offspring size and climate across the main part of the species range occupied by the WV and EV clades. We note, however, that in several cases where two samples from contrasting environments were obtained within the same primary study (samples 4 vs. 5 in French Pyrenees; 24 vs. 25 in the east of European Russia—“Appendix”; southern Sweden vs. northern Sweden—Uller and Olsson 2003), hatchling mass was higher in colder climates. At the same time, hatchlings from females coming from higher vs. lower latitudes in West Siberian plain (samples 30–32 vs. 33–36—“Appendix”), and higher versus lower altitudes in northern Spain (Rodríguez-Díaz and Braña 2012), did not differ in their mass. Thus, the available data on geographic variation of hatchling mass in *Z. vivipara* provide no unequivocal support for Prediction 6. In lizards in general the predicted pattern is really predominant (Mathies and Andrews 1995; Rohr 1997; Qualls and Shine 2000; Wapstra and Swain 2001; Caley and Schwarzkopf 2004; Du et al. 2010). The opposite pattern, smaller hatchlings at colder climates, has been reported by far less often (Sinervo 1990; Li et al. 2011); moreover, in hot and dry regions, the latter pattern can in fact be consistent with the underlying theoretical models (Parker and Begon 1986; Roff 1992; Johnston and Leggett 2002) because the main constraint on juvenile growth is water deficiency rather than thermal opportunities (Díaz et al. 2012; see also Mateo and Castanet 1994). Noteworthy, for *Z. vivipara* a higher hatchling size in drier versus more humid habitats located within 10 km around samples 11–13 (Fig. 1) was reported (Lorenzon et al. 2001).

### Lineage

Our study revealed significant effects of evolutionary lineage on mean hatchling mass. Whereas no consistent differences were found between the two (sister) viviparous clades, such differences occurred between the viviparous and the oviparous clades, and between WO and EO clades. Although this pattern is concordant to differing extent of egg retention, hatchlings of WO clade are only slightly heavier than those of viviparous populations (Table 3). This small inconsistency may be due to phylogenetic history: oviparity of WO clade is likely a reversal from viviparity (Surget-Groba et al. 2006).

For RCM, the effect of lineage was weak and indistinguishable from the effect of reproductive mode. For female SVL and clutch size, no significant effect of lineage was found when climatic vectors are considerd simultaneously. The above results suggest that the lineage as such (i.e. separated from the effects of the extent of egg retention and climate) have a rather moderate impact to shaping geographic variation of reproductive strategies in *Z. vivipara*. In another lacertid lizard, *Psammodromus algirus*, two mt-DNA lineages differed in mean incubation time but not in offspring size, fecundity and maternal size (Díaz et al. 2012). A weak effect of phylogeny on geographic variation of life-history traits was also reported for a widespread iguanian lizard, *Sceloporus undulates* (Niewiarowski et al. 2004). In contrast, the variation in reproductive and other life-history traits *among* species of lacertid lizards included a substantial phylogenetic component (Bauwens and Díaz-Uriarte 1997).

### Co-variation of Offspring Number, Offspring Size, and Maternal Size

Among viviparous populations the pattern and extent of geographic correlations among hatchling mass, clutch size and maternal SVL is very similar to that among individual females within populations, thus following Prediction 7. That is, despite a huge geographic range, this inter-population variation is unlikely to be associated with a considerable evolutionary divergence. The patterns of covariation within and between populations become less similar when including oviparous samples. This is because the higher hatchling mass of oviparous females versus viviparous females is NOT in line with the slightly positive maternal size-offspring size correlation found within populations (positive factor loadings of the both traits on PC1, Table 4; see also Kuranova and Yartsev 2012). Another study which addressed correlations among reproductive traits in a squamate species revealed a stronger discrepancy between the within-population and among-population patterns, the discrepancy having resulted from a pronounced and obviously adaptive divergence in offspring size (Gregory and Larsen 1993, 1996).

### Final Remarks

As was mentioned in the Introduction, for several trait-climate relationships the prevailing adaptive hypothesis and the prevailing plasticity-related hypothesis predict opposite patterns (Table 5). Further, patterns of co-variation among traits within and among populations are expected to be similar when geographic variation is mainly due to plasticity or stochastic genetic processes, but these are likely to differ when a considerable adaptive divergence has occur (cf: Merilä and Björklund 1999; Revell et al. 2007). Even though this association between a pattern and the underlying mechanism is likely rather than strictly obligatory, it is striking that all three independent patterns revealed in *Z. vivipara* suggest a plasticity-related causation (Table 5). Common garden and transplant experiments on this species (Sorci et al. 1996; Sorci and Clobert 1999; Lorenzon et al. 2001) revealed predominantly plastic responses of life-history traits to variation in physical environments. Taken together, these findings suggest that the reproductive strategy of *Z. vivipara* generally exhibits evolutionary conservatism, thus inderctly supporting our principal prediction.

**Table 5:** The prevailing hypotheses which explain different patterns of relationships between phenotypic traits and climatic parameters, and among phenotypic traits in intraspecific geographic variation of reptiles

Trait	Plastic response	Adaptive response
Body size (SVL)	Bergmann’s cline (Prediction 4, confirmed)	Converse Bergmann’s cline
Reproductive output (adjusted clutch size and RCM)	Lower values in colder climates (Predictions 5a, 5b, confirmed)	Higher values in colder climates
Offspring size	?	Higher values in colder climates (Prediction 6)
Pattern of among-population correlations among traits	Similar to the pattern within populations (Prediction 7, confirmed)	Differ from the pattern within populations

See “Introduction” and “Discussion” sections for details

The only pattern of considerable evolutionary divergence in reproductive traits revealed in this study is a decrease in offspring size in a series of three population groups (clades) which represent progressive stages of egg retention. The terminal point of this series, viviparous populations, exhibits the lowest hatchling mass among 64 studied species of European lacertids (Table 1 in In den Bosch and Bout 1998; “Appendix” in this paper), including species with clearly smaller female sizes. Note that many viviparous populations also exhibit quite high mean values of relative clutch mass (60–80 %, “Appendix”) which are among the largest in lizards (Van Damme et al. 1989). Such an association of a high total reproductive investment with a low investment per individual offspring is of particular interest. Classical life-history theory (Smith and Fretwell 1974) modeled these two parameters as independent. However, their negative correlation is predicted by a more recent model (Winkler and Wallin 1987), and such an evolutionary link was found in a few empirical studies (Caley et al. 2001 and references therein). Therefore, even though the evolutionary decrease of offspring size in *Z vivipara* corresponds well to the ‘volume constraint hypothesis’, some additional factors—likely related to an increased selection for fecundity (Einum and Fleming 2000)—might have contributed to this trend. Exploring this complex problem seems to be a promising point for further evolutionary studies on this interesting species.

Another worthy direction of future research is a direct testing of our hypothesis about an evolutionary conservative reproductive strategy in *Z. vivipara*. Long-term common garden experiments (Ferguson and Talent 1993) involving populations which exhibit contrasting phenotypes could estimate to what extent the observed differences are due to proximate effects of environment. Specifically, the differences in female body size between the two oviparous clades, or among the most divergent viviparous populations (i.e. samples 39 and 43) are interesting targets for such studies.

## Appendix

See Table 6.

**Table 6:** Reproductive characteristics for 44 geographically distinct populations of the lizard *Zootoca vivipara*

Study sample (see Fig. 1 for locations)		Clade	Female SVL (mm)					Clutch size					Mean newborn mass (mg)					RCM (%)			Source
ID	Sample details		N	Min	Max	Mean	SD	N	Min	Max	Mean	SD	N	Min	Max	Mean	SD	N	Mean	SD	
1	N Spain, Asturias 1	WO		43.0	63.0	53.0					6.20					210					Bauwens and Diaz-Uriarte (1997)
2	N Spain, Asturias 2	WO	14	44.5	58.2	52.2	3.70	14	2	8	5.30	1.70						12	45.2	9.9	Brana et al. (1991)
3	N Spain, Basque Country	WO	10	50.0	60.5	55.5	2.90	9	5	8	6.50	1.20						8	48.8	5.9	Brana et al. (1991)
4	French Pyrenees, Gabas	WO	32			57.8	3.90	32	3	9	4.70	1.30	10	187	243	215	20.0	29	49.0	11.0	Osenegg (1995)
5	French Pyrenees, Luvie	WO	40			55.2	2.70	32	2	7	4.60	1.30	17	125	230	191	28.0	40	47.0	10.0	Osenegg (1995)
6	S Austria, oviparous	EO	14	55.0	68.0	60.6	3.46	14	4	7	5.40	1.09	13	183	346	277	42.1				Lindtke et al. (2010)
7	S Austria, viviparous	WV	20	58.0	76.0	64.5	4.53	20	3	10	6.50	1.64	15	163	229	201	19.3				Lindtke et al. (2010)
8	S England	WV	50	50.4	66.5	58.5	3.70	50	3	11	7.74	1.61									Avery (1975)
9	NW France, Paimpont	WV	53	49.8	69.1	59.1	4.03	53	2	9	6.51	1.55				181		52	63.4		Pilorge et al. (1983)
10	S France, Besse	WV	106	46.9	67.9	59.2	4.57	106	2	11	5.80	1.75				163		185	63.6		Pilorge et al. (1983)
11	S France, Cevennes 1 (CCML)	WV	15			59.6	3.52	15			6.60	2.29	99			168	18.14	15	56.0	14.0	Pilorge (1987)
12	S France, Cevennes 2 (CMB)	WV	15			55.2	3.47	15			4.33	1.72	67			179	20.45	15	40.0	11.0	Pilorge (1987)
13	S France, Cevennes 3 (CPCE)	WV	15			57.1	3.39	15			4.60	1.64	69			181	17.41	15	42.0	11.0	Pilorge (1987)
14	Switzerland, Berner Oberland	WV	19	56.0	71.0	61.8	4.80	19	3	11	6.32	2.50				200			49.0		Cavin (1993)
15	Belgium, Kalmthout	WV	100	45.5	63.6	53.0	3.60	100	2	7	4.35	0.98	104			178		15	70.6	18.9	Bauwens and Verheyen (1987)
16	E Germany, Schkeuditz	WV	26	55.0	71.0	63.2	4.31	26	5	15	9.77	2.32	17	150	214	179	19.1	3	82.0	28.1	Hofmann original data
17	N Sweden	WV	18			63.6					5.21										Uller and Olsson (2003)
18	S Sweden	WV	70			59.0					6.20		70	138	265	206	26.863				Uller and Olsson (2005)
19	Poland,	WV	10	51.0	65.0	58.2	4.18	10	5	13	9.10	2.42									Juszczyk (1974)
20	S Serbia	WV	24			60.0		24	4	9	6.44	1.5273	20			186	15.556				Crnobrnja-Isailović and Aleksić (2004)
21	lowland Ukraine	EV	22	49.0	71.5	61.5	7.05	22	5	16	9.36	2.94									Zinenko original data
22	Novgorod Region	EV	8	53.2	63.2	57.6	3.50	8	4	8	4.88	1.46									Orlova original data
23	Moscow Region	EV	8	55.0	64.3	60.0	3.45	8	5	9	6.63	1.51									Orlova original data
24	Middle Volga area	EV	20	56.0	68.0	61.7	3.51	20	4	14	8.60	2.48	9	157	223	189	21.6				Eplanova original data
25	Perm Region, North	EV	16	54.0	68.0	59.6	4.27	16	4	9	6.25	1.44	16	179	254	219	18.6				Eplanova original data
26	Perm Region, middle 1	EV	9	50.0	65.0	57.4	4.45	9	2	9	5.22	2.11									Eplanova original data
27	Perm Region, middle 2	EV	36	52.0	69.0	60.2	4.52	36	3	10	6.06	1.76									Orlova original data
28	NE of European Russia	EV	12	55.0	77.0	64.8		18	3	10	6.40	1.82									Anufriev and Bobretsov (1996)
29	W Siberia, northern taiga	EV	10	47.5	68.6	61.5	5.97	10	4	9	6.30	1.42									Shamgunova, Orlova original data
30	W Siberia, middle taiga	EV	24	52.0	73.0	61.8	4.59	24	3	11	6.33	2.39	5	183	214	200	13.5	4	52.3	6.9	Shamgunova original data
31	W Siberia, middle taiga, North	EV	11	60.0	73.7	63.7	4.01	11	4	8	6.09	1.14									Shamgunova original data
32	W Siberia, S taiga	EV	30	53.9	72.4	61.8	4.42	30	2	10	6.47	2.05	24	139	232	190	25.1				Kuranova, Bulakhova original data
33	W Siberia, subtaiga 1	EV	20	45.6	74.9	61.4	6.16	20	3	11	6.95	2.19	5	180	208	189	11.8				Kuranova, Bulakhova original data
34	W Siberia, subtaiga 2	EV	138	46.2	71.5	60.0	4.45	138	2	14	6.24	1.98	54	140	298	207	28.3	17	76.2	14.3	Kuranova, Bulakhova original data
35	W Siberia, subtaiga 3	EV	52	50.7	69.7	60.2	3.56	52	4	9	5.58	1.23	24	138	260	194	31.5	3	63.4	6.5	Kuranova, Bulakhova original data
36	W Siberia, subtaiga 4	EV	31	50.8	69.8	60.1	4.52	31	2	10	6.45	2.00	16	146	222	197	21.289	6	47.6	15.9	Kuranova, Bulakhova original data
37	W Siberia, Kuznetsky Alatau, North	EV	24	52.0	67.6	61.4	3.43	24	3	10	6.00	1.57									Orlova et al. (2005)
38	W Siberia, Kuznetsky Alatau, West	EV	16	59.0	76.0	66.0	4.61	16	4	8	6.13	1.26									Kuranova original data
39	W Siberia, Kuznetsky Alatau, East	EV	12	62.4	74.3	70.3	3.98	12	7	10	8.50	1.09									Kuranova and Bulakhova original data
40	W Siberia, N Altai	EV	12	46.8	69.1	57.8	6.40	12	3	13	6.92	2.64									Kuranova and Bulakhova original data
41	W Siberia, NE Altai, low elevations	EV	21	53.4	75.0	61.1	5.84	21	3	12	6.24	2.77									Yakovlev original data
42	W Siberia, NE Altai, high elevations	EV	31	50.0	71.0	63.3	4.35	31	4	10	7.06	1.67									Yakovlev original data
43	NE Kazakhstan, Markakol	EV	37	46.0	68.0	54.7	4.78	37	2	8	4.95	1.27									Orlova original data
44	NE China, Sinvu	EV	26	55.9	71.0	64.5	3.52	26	3	12	7.11	2.47	26	110	240	170	30.0				Liu et al. (2008)

Clades (after Surget-Groba et al. 2006): *WO* western oviparous, *EO* eastern oviparous, *WV* western viviparous, *EV* eastern viviparous
